# Molecular docking between human TMPRSS2 and SARS-CoV-2 spike protein: conformation and intermolecular interactions

**DOI:** 10.3934/microbiol.2020021

**Published:** 2020-09-24

**Authors:** Mushtaq Hussain, Nusrat Jabeen, Anusha Amanullah, Ayesha Ashraf Baig, Basma Aziz, Sanya Shabbir, Fozia Raza, Nasir Uddin

**Affiliations:** 1 Bioinformatics and Molecular Medicine Research Group, Dow Research Institute of Biotechnology and Biomedical Sciences, Dow College of Biotechnology, Dow University of Health Sciences, Karachi-Pakistan; 2 Department of Microbiology, University of Karachi, Karachi-Pakistan; 3 Faculty of Computer Science, IBA, Karachi-Pakistan

**Keywords:** SARS-CoV-2, COVID-19, spike protein, TMPRSS2, molecular docking

## Abstract

Entry of SARS-CoV-2, etiological agent of COVID-19, in the host cell is driven by the interaction of its spike protein with human ACE2 receptor and a serine protease, TMPRSS2. Although complex between SARS-CoV-2 spike protein and ACE2 has been structurally resolved, the molecular details of the SARS-CoV-2 and TMPRSS2 complex are still elusive. TMPRSS2 is responsible for priming of the viral spike protein that entails cleavage of the spike protein at two potential sites, Arg685/Ser686 and Arg815/Ser816. The present study aims to investigate the conformational attributes of the molecular complex between TMPRSS2 and SARS-CoV-2 spike protein, in order to discern the finer details of the priming of viral spike protein. Briefly, full length structural model of TMPRSS2 was developed and docked against the resolved structure of SARS-CoV-2 spike protein with directional restraints of both cleavage sites. The docking simulations showed that TMPRSS2 interacts with the two different loops of SARS-CoV-2 spike protein, each containing different cleavage sites. Key functional residues of TMPRSS2 (His296, Ser441 and Ser460) were found to interact with immediate flanking residues of cleavage sites of SARS-CoV-2 spike protein. Compared to the N-terminal cleavage site (Arg685/Ser686), TMPRSS2 region that interact with C-terminal cleavage site (Arg815/Ser816) of the SARS-CoV-2 spike protein was predicted as relatively more druggable. In summary, the present study provides structural characteristics of molecular complex between human TMPRSS2 and SARS-CoV-2 spike protein and points to the candidate drug targets that could further be exploited to direct structure base drug designing.

## 1. Introduction

The recent pandemic of COVID-19 is the third outbreak of the diseases caused by beta coronavirus in humans, following Severe Acute Respiratory Syndrome (SARS) and Middle Eastern Respiratory Syndrome (MERS) [1]. As of 26th August 2020, over 23 million of global population has been infected with mortality rate of 5% in closed cases [2]. Genetically, etiological agent of COVID-19, SARS-CoV-2, is closely related to SARS-CoV compared to MERS-CoV [3]. Similarly, as in SARS-CoV, Angiotensin Converting Enzyme-2 (ACE2) has been identified as the primary receptor for SARS-CoV-2 spike protein [4],[5]. Whereas MERS-CoV spike protein interacts with the DiPeptidyl Peptidase 4 (DPP4) as the first site of attachment to the host cell [4]. Spike protein of SARS-CoV-2 is 1273 amino acid long protein with two functionally distinct regions, S1 and S2, involved in the attachment and entry of the virus, respectively. SARS-CoV-2 entry in the host cell is mediated by proteolytic cleavage of its spike protein, a process dubbed as priming. Recently, human Transmembrane Protease Serine 2 (TMPRSS2) and potentially furin have been shown to carry out the priming of the SARS-CoV-2 spike protein by generating two distinct fragments of the viral spike protein, S1/S2 and S2' [6],[7].

Recently, co-crystal structure of SARS-CoV-2 spike protein complexed with ACE2 receptor has been resolved unraveling the finer details of intermolecular interactions [5]. The ACE2-SARS-CoV-2 complexes not only indicate the potential of cross species transmission but also open a window for designing and/or screening of disruptor molecules that could potentially inhibit the attachment of the virus with the host cells [5]. However, no complex structure of SARS-CoV-2 spike protein with TMPRSS2 has been resolved to date. Moreover, the molecular structure of human TMPRSS2 protein is also not known. Resultantly, structural details of intermolecular interactions between SARS-CoV-2 and TMPRSS2 are largely unknown. Although, like many other protease inhibitors [8], TMPRSS2 inhibitor has been suggested and/or shown to antagonize the entry of the virus into the host cells [6]. This study aims to investigate the molecular complex between TMPRSS2 and SARS-CoV-2 spike protein using an array of bioinformatic tool. The findings not only provide structure-function relationship of human TMPRSS2 but also predict sites of molecular interactions between TMPRSS2 and SARS-CoV-2 spike protein. This could lead to the development and/or directed screening of disruptor and/or inhibitor molecules, targeting the viral entry into the host cell.

## 2. Methodology

### 2.1. Data mining for structures

Protein sequence of human TMPRSS2 (O15393) was retrieved from UniProt and subjected to PDB Blast to identify the homologous structure on the basis of query coverage and sequence identity. Atomic coordinates of SARS-CoV-2 spike protein (PDBid: 6VSB) and Hepsin (PDBid: 1Z8G) were retrieved from RCSB protein data bank [9],[10],[11]. Probability of the protein crystallization for TMPRSS2 was predicted using XtalPred server [12].

### 2.2. Molecular modelling

N-terminal region (1–148) of TMPRSS2 including LDL-receptor class A domain was modeled using I-TASSER due to the unavailability of template with sufficient homology [13]. Since, hepsin molecule was found to share noticeable homology with the SRCR and peptidase S1 domains of TMPRSS2, a full length model of TMPRSS2 were later developed using Modeller 9.16 taking model developed by I-TASSER and hepsin (PDBid: 1Z8G) as templates [14]. Full length model of TMPRSS2 was further refined for Gibb's free energy and conformation of the loops. The structural quality of the model was assessed by MolProbity for Ramachandran Plot [15] and ProSA [16]. Finally, the full length model was superimposed over template (PDBid: 1Z8G), plasma kallikrein (PDBid: 5TJX) and root mean square deviation in carbon alpha back bone was measured in Å using Swiss PDB Viewer v4.1.0 [17]. Missing regions in the structurally resolved SARS-CoV-2 spike protein (PDBid: 6VSB) were also modelled using Modeller 9.16. Molecular dynamic simulation for TMPRSS2 model was carried out in water as solvent for 25000 picoseconds using GROMACS under default parameters [18] and RMSD in the protein architecture was measured in nm.

### 2.3. Molecular docking

HADDOCK 2.2 webserver was used to conduct molecular docking between SARS-CoV-2 spike protein and TMPRSS2 [19]. The input includes atomic coordinates of SARS-CoV-2 spike protein (PDBid: 6VSB) with gaps being filled and constructed full length molecular model of TMPRSS2. Two separate docking simulations were run for each cleavage site of the viral spike protein. Reported cleavage sites [6] on spike protein were defined as active residues for SARS-CoV-2, whereas substrate binding sites and catalytically active sites were recognized as active residues of TMPRSS2. HADDOCK congregated all docking simulations into clusters and ranked them according to the HADDOCK score which is the function of linear combination of Van der Waals energy, electrostatic energy, desolvation energy, restraint violation energy and buried surface area. The cluster with least HADDOCK score was selected for further assessment. Binding affinity and different types of interactions like charged-charged, charged-polar, charged-apolar, polar-polar, polar-apolar and apolar-apolar were identified using PRODIGY webserver [20]. All structures were visualized using DS visualizer 2016.

## 3. Results

### 3.1. Molecular model of TMPRSS2

**Figure 1.**
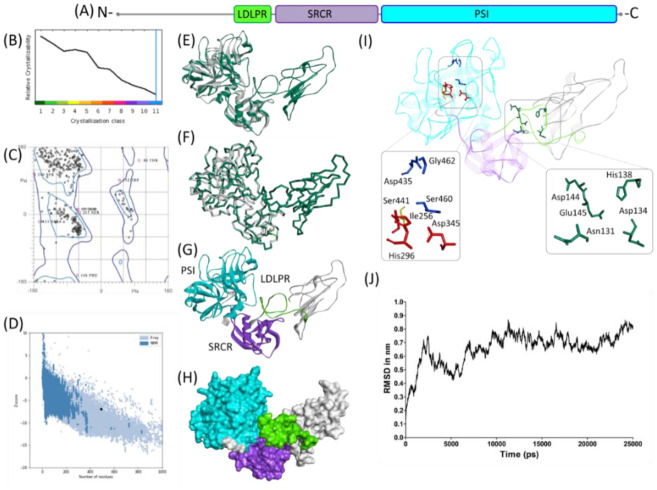
Molecular structure of TMPRSS2. (**A**) Scaled schematic representation of the functional domains of TMPRSS2 protein (**B**) XtalPred analysis where blue line represents the least probability of the crystallization (**C**) Ramachandran plot of the model (**D**) ProSA analysis of the model where the Z score of the model is indicated by black dot, whereas Z scores of resolved structures are shown with dark blue (NMR) and light blue (X-ray) shades. Superimposition of the full length TMPRSS2 model with template in (**E**) ribbon (**F**) Cα backbone conformations. Ribbon conformation (**G**) and surface topology (**H**) of TMPRSS2 structure where domains are coloured differently and labelled at the corresponding positions. (**I**) Functionally important residues are shown in green, blue, red and yellow sticks representing calcium binding sites, substrate binding sites, catalytic sites and proteolytic cleavage site, respectively. (**J**) Molecular dynamic simulation of TMPRSS2 model showing reasonable stability of the molecule after 10000 picoseconds of the simulation run.

Human TMPRSS2 is 492 amino acid long protein with three functional domains: an N-terminal LDL-receptor class A domain (113–148) followed by SRCR (153–246) and finally at C-terminal peptidase S1 domain spanning from 256 to 487 amino acid (Figure 1A). Till now molecular structure of the protein has not been resolved and our XtalPred analysis showed the least possibility for this molecule to be crystalized, potentially due to the high percentage of coiled structure, isoelectric point and surface hydrophobicity (Figure 1B). This may be the reason that since the first report of TMPRSS2 in year 1997, the structure has not been resolved yet by X-ray crystallography [21]. Nevertheless, computational based molecular modelling approaches have evolved since then and come of age in terms of accuracy and reliability with new tools and server being available [13],[14]. Therefore, we used multiple approaches to develop the full-length molecular model of TMPRSS2. The finally selected refined model of TMPRSS2 has 96.32% residues within the allowed regions of Ramachandran plot, which is acceptable considering the N-terminal portion of the protein was predicted to be intrinsically disordered. Secondly, it has been demonstrated rather frequently that many of the resolved structures of the proteins such as USP7 (PDB id: 2F1Z) have more than 20% of the residues outside the allowed region in Ramachandran plot. Moreover, Gibbs Free energy values (−14212.818 KJ/mol) and ProSA Z score (−6.89) both suggest structural reliability of the model (Figure 1C and D).

### 3.2. Molecular structure of TMPRSS2

Full length molecular model of TMRPSS2 has considerable structural homology with the template molecule (PDB: 1Z8G), where the deviation between the Cα backbone of model and template was found as 0.33Å (Figure 1E and F). All three domains, LDL-receptor class A, SRCR and peptidase S1, formed distinct structural units in the molecular model. N-terminal region and LDL-receptor class A of TMPRSS2 were found more or less unstructured (Figure 1G and H). Putative Ca2+ biding residues (Asp134, His138, Asp144, Glu145 and Asn131) were found on a loop linking N-terminal of the protein with SRCR domain. Structurally, SRCR domain comprises an α helix and multiple anti parallel β sheets, potentially stabilized by two disulfide bonds between Cys172-Cys231 and Cys185-Cys241 (Figure 1I). Overall structural conformation of the domain showed uncanny resemblance with the SRCR domain found in MARCO receptor and hepsin [10],[22]. The C-terminal of TMPRSS2 has a large catalytic domain with typical structural features of chymotrypsin family serine proteases [23]. The triad of catalytically active site residues (His296, Asp345 and Ser441) and substrate binding sites (Asp435, Ser460 and Gly462) were found sandwiched between two six stranded β barrels of nearly equal size (Figure 1I). The inter-residual distance between the catalytically active residues ranges from 6.494Å to 9.754Å and spatially very similar to the catalytic residues of hepsin (PDB: 1Z8G) and plasma kallikrein (PDB: 5TJX) (Figure S1A). The inter-residual distance between substrate binding sites ranges from 7.409Å to 11.765Å (Figure S1B). The globular conformation of the domain is likely be stabilized by four disulfide bonds between Cys244-Cys365, Cys281-Cys297, Cys410-Cys426 and Cys437-Cys465. Moreover, molecular dynamic simulation in water for 25000 picoseconds represents structural stability of the constructed model, as limited deviation in the RMSD in Cα backbone was observed after 10000 picoseconds (Figure 1J). PDB file of the model is made available in supplementary materials Model and Complexes.

### 3.3. Interaction of TMPRSS2 with SARS-CoV-2 Spike protein

**Figure 2.**
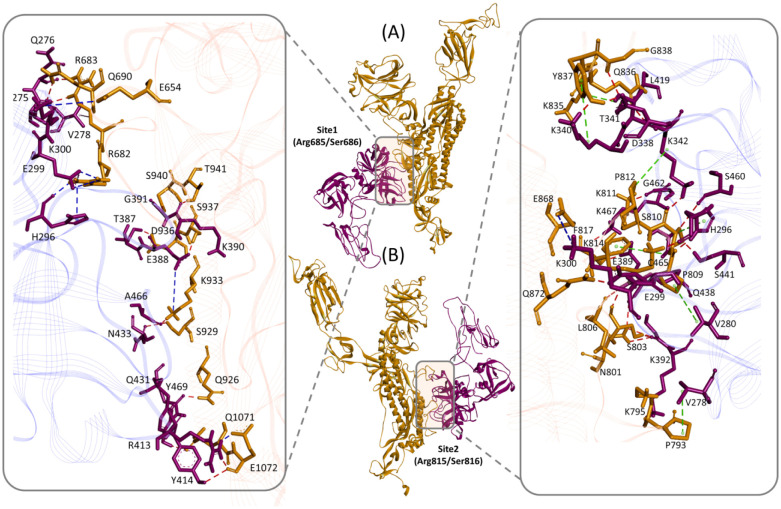
TMPRSS2 and SARS-CoV-2 spike protein Molecular Complex. Ribbon diagram of complexes between TMPRSS2 (magenta) and SARS-CoV-2 spike protein (gold) for (**A**) site1 (Arg685/Ser686) and (**B**) site2 (Arg815/Ser816), residues of TMPRSS2 (magenta sticks) and spike protein (gold sticks) involved in the intermolecular interactions are shown in the respective boxes. PDB files of the complexes are made available in supplementary materials Model and Complexes.

Proteolytic cleavage of the viral spike protein, resulting in the formation of two fragments, S1/S2 and S2' by host TMPRSS2 is pre-requisite for viral entry into the host cell [6]. The precise positioning of the proteolytic cleavage sites has been mapped by sequence comparison and found to be at the junction of Arg685/Ser686 and Arg815/Ser816. The cleavage at the later site results in the production of S1/S2 and S2' fragments, which is necessary for the viral entry into the cells. This provides an excellent basis on which docking simulations could be directed. Thereby, in this study we run an independent docking simulation for each site. The conformation of the complex between TMRPSS2 and SARS-CoV-2 in selected docking pose revealed that both cleavage sites of SARS-CoV-2 spike protein are present at the flexible loops and interacts with one of the β barrel of the catalytic domain of TMPRSS2 (Figure 2). At the first cleavage site (Arg685/Ser686) of the spike protein, His296 of TMPRSS2 formed a hydrogen bond and electrostatic interaction with Arg682 of the spike protein (Table 1) (Figure 2A). Whereas, at the second cleavage site (Arg815/Ser816), out of the three residues of catalytic triad, His296 and Ser441 established hydrogen bond interactions with Pro809, Lys814 and Ser810 of the SARS-CoV-2 spike protein (Table 1) (Figure 2B). Ser810 also formed a hydrogen bond and hydrophobic interaction with Ser460, substrate binding site, and His296, catalytic site of TMPRSS2 (Table 1) (Figure 2B). Since the functionally important residues of TMPRSS2 interact with the amino acids that immediately flank the cleavage site, this raises a possibility that upon interaction with the viral spike protein, the later may undergo conformational changes that may bring Arg685/Ser686 and Arg815/Ser816 of the SARS-CoV-2 spike protein in line with the active site cleft of TMPRSS2. Nevertheless, Ser441 of TMPRSS2, that has been demonstrated as the most critical residue for the proteolytic cleavage of viral spike protein [24],[25], were found interacting with several flanking residues of cleavage site found in SARS-CoV-2 spike protein (Table 1) (Figure 2B). This represents the importance of neigbouring residues in the establishment of molecular complex between TMPRSS2 and SARS-CoV-2 spike protein. Furthermore, involvement of charged residues in most of the intermolecular interactions and binding affinity values (−13.8 Kcal/mol) represent the reliability of the complex in terms of structural conformation (Table 1).

**Table 1:** Comparison of intermolecular interactions between TMPRSS2 and two different cleavage sites of SARS-CoV-2 Spike protein.

Nature and Properties of Interactions	Cleavage Site 1 (Arg685/Ser686)		Cleavage Site 2 (Arg815/Ser816)	
	TMPRSS2	SARS-CoV-2 Spike Protein	TMPRSS2	SARS-CoV-2 Spike Protein
Intermolecular Hydrogen Bonds	ARG413	GLU1072	HIS296	PRO809
	GLU299	ARG682	LYS340	TYR837
	THR387	ASP936	LYS342	GLN836
	ARG413	GLU1072	LYS342	SER810
	TYR414	GLU1072	LYS392	LYS795
	TYR469	GLN1071	GLN438	SER803
	HIS296	ARG682	GLN438	LEU806
	VAL275	ARG683	SER441	SER810
	GLN276	ARG683	LYS467	GLN935
	LYS300	GLN690	GLY391	ASN801
	GLN431	GLN926	GLU389	ASN801
	ASN433	SER929	GLY391	ASN801
	ALA466	LYS933	GLY391	SER803
	GLU388	SER937	GLY462	LYS811
	LYS390	THR941	HIS296	LYS814
	GLY391	SER940	ASP338	TYR837
	ARG413	GLU1072	THR341	GLY838
	GLU299	ARG682	GLU299	GLN872
			GLU389	GLN935
			SER460	SER810

Intermolecular Electrostatic Interactions	Lys300	GLU654	LYS300	GLU868
	GLU299	ARG682		
	GLU299	ARG682		
	GLU388	LYS933		
	HIS296	ARG682		
	ARG413	GLU1072		

Intermolecular Hydrophobic Interactions	VAL278	ARG683	HIS296	SER810
			VAL278	PRO793
			VAL280	PRO809
			LYS342	PRO812
			LEU419	LYS835
			CYS465	PHE817
			LYS340	TYR837

Charged-Charged	9		11
Charged –Polar	21		32
Charged-Apolar	17		27
Polar-Polar	12		11
Polar-Apolar	16		28
Apolar-Apolar	3		13
Binding Affinity	-9.8 Kcal/mol		-13.8 Kcal/mol
Kd at 25°C	6.8 × 10^−8^		7.1 × 10^−11^
Kd at 37°C	1.3 × 10^−7^		1.8 × 10^−10^

## 4. Discussion

Human TMPRSS2 is a 70kDa protein, a member of large superfamily of serine protease, mainly expressed in prostate, colon, stomach, eye and salivary gland [26],[27]. In prostate gland its expression is regulated by androgens and found overexpressed in prostate carcinoma [28]. Physiologically, the protein is important in the functioning of epithelial sodium transport [29] and angiogenesis [30]. In addition, TMPRSS2 importance has been demonstrated in relation to the entry of influenza virus [24], SARS-CoV [31], parainfluenza virus [32], MERS-CoV [33] and SARS-CoV-2 [6]. Cleavage sites of SARS-CoV-2 spike protein for TMPRSS2 action have been mapped, but the complex structure of SARS-CoV-2 spike protein and TMPRSS2 has not been resolved. An investigative flank of the unpublished study (DOI: 10.1101/2020.02.08.926006) attempted to address the same issue, however, focusing on the development of peptidyl analogue targeting merely catalytic triad of TMPRSS2 using partial model of the molecule. Additionally, details regarding the interaction between the viral spike protein and TMPRSS2 have not been resolved at the residual level and/or for both cleavage sites.

Given the apparent difficulty in attaining the crystal structure of TMPRSS2 (Figure 1B) and the lack of structural information of the protein opens a window for computational modelling and molecular docking to predict the breadth of intermolecular interactions between SARS-CoV-2 spike protein and TMPRSS2. Therefore, we have constructed full length model of TMPRSS2 showing distinct localization of all three functional domains. It is important to mention that TMPRSS2 share 42.5% query coverage with plasma kallikrein (PDB id; 5TJX) but it covers only the trypsin domain of the protein, in comparison hepsin molecule (PDBid: 1Z8G) though share 33% sequence identity with TMPRSS2 but it covers both SRCR and trypsin domains of the target protein. Moreover, both plasma kallikrein and hepsin share comparable sequence identity with trypsin domain of TMPRSS2. Accounting this, hepsin molecule was taken as template for construction of full-length model of TMPRSS2. The C-terminal peptidase S1 domain of TMPRSS2 is expectedly involved in the interaction with SARS-CoV-2 spike protein. Both substrate binding and catalytic sites residues of TMPRSS2 interact with the cleavage sites and/or immediate flanking residues of SARS-CoV-2 spike protein. Nature of the neighbouring amino acids to the active site of TMPRSS2 and cleavage sites of SARS-CoV-2 provides important clues for the design of targeted inhibitors and/or peptidyl disruptors. Several protease inhibitors have been proposed by means of virtual screening [34] and have shown efficacy against SARS-CoV-2 infection [6]. The findings of the present study in relation to the diversity of the nature of intermolecular interactions and biophysicochemical properties of entailing amino acids may in turn facilitate structure-based drug designing for the more efficient peptidyl antagonists against COVID-19. Peptidyl inhibitors have shown to efficiently inhibit EBNA1 dimerization [35], protein-protein interactions of coiled-coiled transcription factors like Bcl-2 proteins, MDM2/MDMX, HIVgp41 [36] and human thymidylate synthase [37]. In addition, the present study further points to the key residues for the subsequent investigations like site directed mutagenesis and peptide array studies to discern importance of potentially other residues in the priming of the viral spike protein. Recently, we have reported that allelic variants of human ACE2 receptor that flanks the key interacting amino acids for SARS-CoV-2 spike protein may hamper viral attachment to the host [38]. Therefore, the present study could also be advanced in relation to explore the effect of natural polymorphism found in human TMPRSS2 on priming of SARS-CoV-2 spike protein. Recently, paralogue of TMPRSS2, TMPRSS4, and furin has also been demonstrated for its involvement in viral invasion within human cell. This study could provide a template for similar studies involving potential binding between TMPRSS4 and SARS-CoV-2 Spike protein [39],[40].

Since the present study is solely based on computational analysis, thereby it inherently holds some noteworthy limitations. For example, increasing the timing of MD simulation to more than 200ns may further validate the stability of the TMPRSS2 model. Consistently, step wise comparison of catalytic triad of TMPRSS2 with other serine proteases may further represent the validity of the model. Molecular dynamic simulation of TMPRSS2 and SARS-CoV-2 spike protein complex could be carried out to ascertain the conformation and intermolecular interactions in the complex. More importantly, empirical validation employing approaches like site directed mutagenesis and peptide array is essentially warranted, for which our findings may be used to prioritize the key regions.
