# Supplementary figures and images for: Molecular docking between human TMPRSS2 and SARS-CoV-2 spike protein: conformation and intermolecular interactions
Source: AIMS Microbiol. 2020 Sep 24;6(3):350–60. doi: 10.3934/microbiol.2020021 (PMC7535071; doi:10.3934/microbiol.2020021)

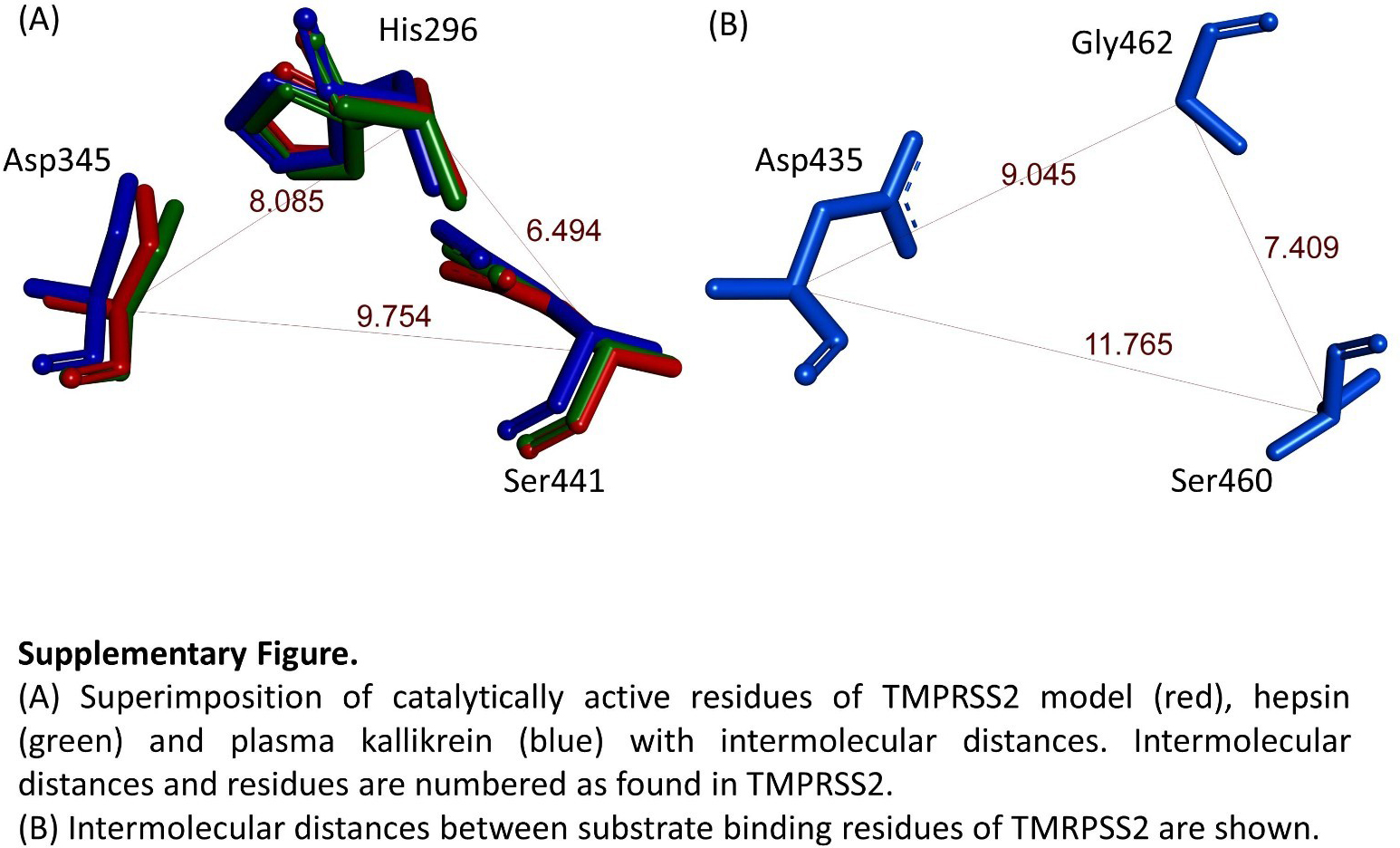

Supplement: Supplementary file 1 [file microbiol-06-03-021-s001.jpg]
